# Ultrasound-Guided Synovial Biopsies of Wrists, Metacarpophalangeal, Metatarsophalangeal, Interphalangeal Joints, and Tendon Sheaths

**DOI:** 10.3389/fmed.2019.00002

**Published:** 2019-01-21

**Authors:** Ilias Lazarou, Stephen Gerard Kelly, Laurent Meric de Bellefon

**Affiliations:** ^1^Department of Rheumatology, University Hospitals of Geneva, Geneva, Switzerland; ^2^Department of Rheumatology, Barts Health NHS Trust, Mile End Hospital, London, United Kingdom; ^3^Department of Rheumatology, Cliniques Universitaires Saint-Luc, Université catholique de Louvain, Brussels, Belgium

**Keywords:** ultrasound-guided synovial biopsy, wrist, metacarpophalangeal, metatarsophalangeal, proximal interphalangeal

## Abstract

Ultrasound-guided synovial biopsy (UGSB) is a minimally-invasive procedure which allows quality synovial tissue retrieval. In this article, we will discuss overarching principles of the procedure performed in wrists, metacarpophalangeal (MCP), metatarsophalangeal (MTP), interphalangeal joints (IP), and tendon sheaths, including basic sonoanatomy, entry site and biopsy technique, as well as special considerations for each structure whenever relevant.

## Introduction

Synovial tissue analysis is fundamental for basic research on inflammatory arthritis (IA) pathobiology, and in the quest of biomarkers of response to treatment (1–5). The advance of musculoskeletal ultrasound (US) over the past decades as a reliable imaging tool has led to development of US-guided synovial biopsy (UGSB) techniques, using a portal-and-forceps or semi-automatic biopsy needle (6, 7). UGSB is increasingly used to harvest synovial tissue in the setting of clinical trials (8) and clinical routine. UGSB is a well-tolerated procedure (6), has a favorable safety profile and can be repeated on a serial basis as it does not alter subsequent clinical or US joint assessments (9). Recommendations for minimal standards for reporting (10) and for sample retrieval for small joint biopsies (11) have already been published and will not be discussed in this review, which focuses on technical aspects of the procedure in wrists and small joints.

Prerequisites are sufficient musculoskeletal US experience and US-guided procedures or specific training in UGSB. A learning curve certainly exists with regards to procedure duration and tissue quality-RNA yield. It is strongly recommended to keep records of these three parameters as well as patient tolerance data as an index of quality control. The percentage of graded samples and RNA yield should be coupled to disease activity measures, US findings of the biopsied joint and personal experience (a highly inflamed large joint will probably provide more tissue and RNA in the hands of an experienced operator).

## Prior to the Procedure

Patient eligibility should be verified and include but not limited to absolute and relative contraindications to UGSB, such as active skin infection and anticoagulant/antiplatelet treatment, respectively. A written informed consent should be obtained before the procedure and documented in patient notes. We usually consent patients for the most common or severe complications such as wound and joint bleeding, swelling, pain, neurovascular, and tendinous-ligament damage, wound and joint infection, thrombophlebitis and vein thrombosis.

The choice of the area to biopsy (joint and specific joint compartment) depends on local (synovial thickening-ST and Power Doppler -PD) and global patient factors (such as patient's preference and comorbidity). According to the previously published algorithm for biopsy site selection, the ideal candidate joint will demonstrate significant ST with high grade PD (6).

An appropriate clean room must be available for the procedure. Any room suitable for patient care as per local policies may be used, as long as adequate space is provided for the patient, the operator and assistant, the US machine, and the procedure tray.

A checklist of the required material and standardized operating protocol can be found on synovialbiopsy.com (see Table 1) and should appear on the report form. The choice between a portal-and-forceps and a needle approach depends on the joint (needle biopsy is adequate for most small joints) and personal preference. Their differences and similarities have been discussed elsewhere (10). The most common biopsy needle calibers used for the wrist and small joints are 16-Gauge (5 French) and 18-G. Sterile gel use is optional. Alternatively, a chlorhexidine solution can be used as a contact medium.

**Table 1:** Pre-procedure checklist.

Signed consent form	Sample container	Sterile swabs
Biopsy needles	10 mls / 20 mls syringes	Sterile gown and gloves
21G/19G needles	Antiseptic solution	Sterile drapes
Procedure pack	Local anesthetic	Sterile ultrasound sheath
Face mask and hair cover	Non-adhesive dressing	Sterile gel (optional)

After the procedure, compression of the entry site is followed by application of a small adhesive dressing and a quick neuro-vascular assessment of the hand or foot. Contact details of the department, as well as a prescription for painkillers on demand must be handed to the patient before discharge.

## Wrist Biopsy

Together with the knee, the wrist is one of the most commonly biopsied joints (10), due to its relative ease of access and prevalence of involvement in IA especially rheumatoid arthritis (RA).

**Figure 1:**
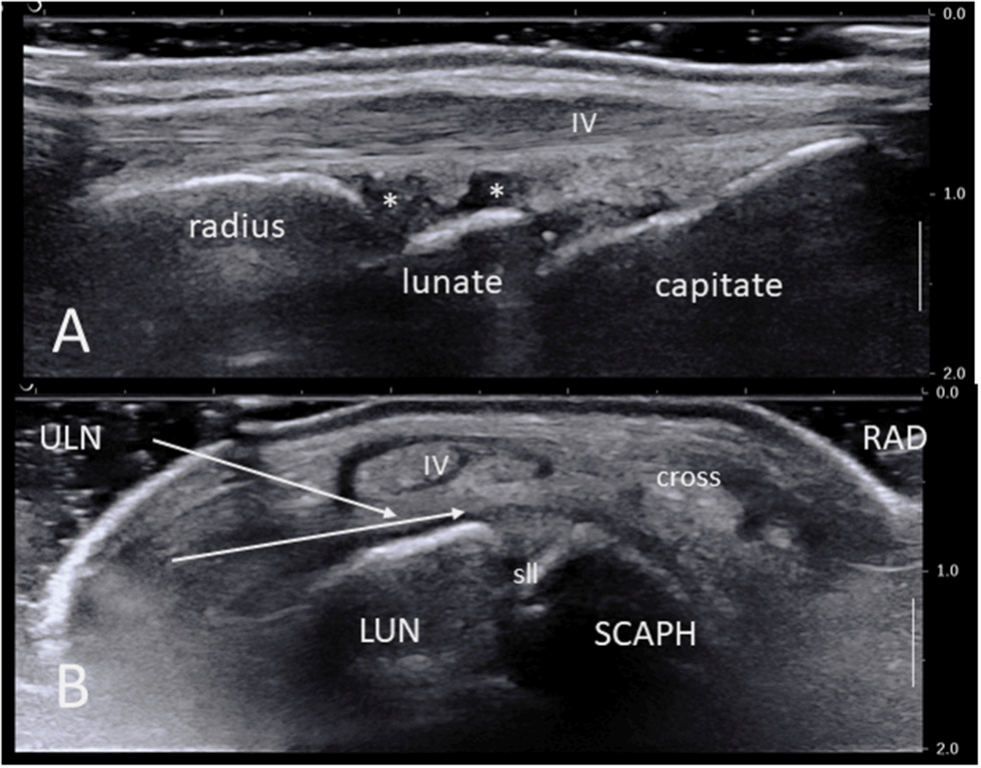
**(A)** Ultrasound long-axis scan of a normal wrist joint using a high frequency linear transducer. Note the radius, lunate, and capitate bone surfaces, the synovial recesses (asterisks), the IV extensor tendon compartment and its retinaculum. **(B)** Short axis ultrasound scan of a normal right wrist. The area of maximal synovial thickening usually lies posterior and radially to the scapho-lunate junction. White arrows show the two most common needle paths between extensor tendons compartments V and IV and VI and V. IV: fourth extensor tendon compartment; cross: crossing point between compartments III and II; LUN, semi-lunate bone; RAD, radial side; SCAPH, scaphoid bone; sll, scapho-lunate ligament; ULN, ulnar side.

Normal sonoanatomy of the wrist is shown in Figure 1A. It is important to identify the various structures and area of interest before the procedure, bearing in mind that in cases of IA this might be challenging due to bone erosions and osteophytes and loss of normal architecture. A suitable path for the needle should thus be planned beforehand.

The patient must be comfortable, supine or recumbent at 45°, with the hand placed on a table next to her/his bed or seat, palm downwards. An absorbent pad is placed on the table. Significant arm elevation and/or abduction should be avoided and the bed should be moved instead. After wearing a mask and hear cover, the operator proceeds to hand disinfection and then wears a sterile gown and sterile gloves. Wearing a second pair of gloves that is discarded after disinfection is preferred by many, even though there is –to our best knowledge- no evidence to support fewer infectious complications in the setting of UGSB. Patients are asked to rest their arm on their elbow and then the hand, wrist and forearm are prepped with a disinfectant soap or solution with emphasis on the entry site. Once clean, the patient is asked to lift her/his arm, then sterile drapes are positioned on the table and a sterile drape used as a cuff at the mid forearm. The hand is then placed in pronation and gentle flexion (one may use sterile gauzes under the volar side of the wrist). A sterile sheath is placed around the US probe, and 10 ml of lidocaine are aspirated in a syringe.

In general, the most suitable harvesting site lies dorsally (anatomically) and slightly radially to the scapho-lunate junction (Figure 1B). On a short axis US scan this corresponds to the area between the scaphoid, scapholunear ligament and lunate bone at the bottom, and the extensor tendon compartments II-IV overlying the long radio-luno-triquetral ligament on the top. Indeed, this area is often the most significantly thickened in IA, but the ulnar synovial recess (deep to and on the ulnar side of the extensor compartment IV) may also be suitable.

The local anesthetic and biopsy needles are thus most commonly inserted on the ulnar side of the wrist, distally to the ulnar styloid process. The exact entry site will depend on case-specific anatomy, but a suitable path can be found between the extensor carpi ulnaris (ECU) and extensor digiti quinti proprius (EDQ) tendons, or –less often- between the EDQ and extensor digitorum communis (EDC) tendons or even on the volar side of the ECU tendon (although not privileged because of the risk of lesion of the triangular fibrocartilage complex). As with any US-guided procedure, once the needle trajectory has been determined by the operator, the US probe must remain still while the needles are inserted in a longitudinal plane (i.e., following the probe's long axis). The subcutaneous tissue at the entry site is first injected with a small volume of local anesthetic such as lidocaine 1% 1–3 ml. Once the skin is anesthetised (usually within 1–3 min), the tissue layers lying between the skin and joint capsule are injected with a suitable volume of lidocaine, e.g., using a 19G needle. Any synovial fluid should be aspirated before the intra-articular lidocaine injection (to prevent false negative culture results owing to its bactericidal effect and false positive due to preparation and handling) and sent for analysis as indicated. The joint space itself is then injected e.g., with 2–5 ml of 1% lidocaine, which is also helpful in distending the joint and allowing for better identification of the synovial membrane under US once the synovial fluid has been removed.

The biopsy needle should be primed before insertion. Once in the area of interest, the throw is opened and must be visualized on the US scan. It is then positioned with a gentle pressure against the synovium to maximize tissue yield. Likewise, applying mild pressure on the probe might also prove helpful in keeping the throw against the synovial surface. The tip of the biopsy needle should be free to move, i.e., not abut against bone or osteophytes, as the triggering mechanism tends to cause a backwards move of the whole needle due to recoil momentum.

Keeping continuous hand- patient skin contact is preferred by some operators for a better control of the biopsy needle. Some operators opt for use of a portal, while others, including the authors, avoid it, as the path created by the first couple of insertions is considered sufficient. Our experience is that this does not increase the risk of complications and published data on safety using this approach is excellent (6).

Synovial tissue is first harvested from the area overlying the biopsy needle, i.e., in the direction of the probe. The three-dimensional structure of the joint space should be kept in mind: once no more tissue is retrieved from a given area, the synovial membrane distally, proximally, and deeply may be biopsied. This is achieved using the same path, while rotating the needle accordingly once inserted and applying pressure against the membrane as described before.

Bleeding –while rare- is a complication of virtually any invasive procedure. Special care must be given to avoid visible vessels such as the branches of the radial artery lying between the V and IV, and IV and III extensor tendon compartments. These arteries are not inside the synovial membrane, but could in theory be punctured if overzealous pressure is applied on the biopsy needle during triggering.

## MCP and MTP Joints

**Figure 2:**
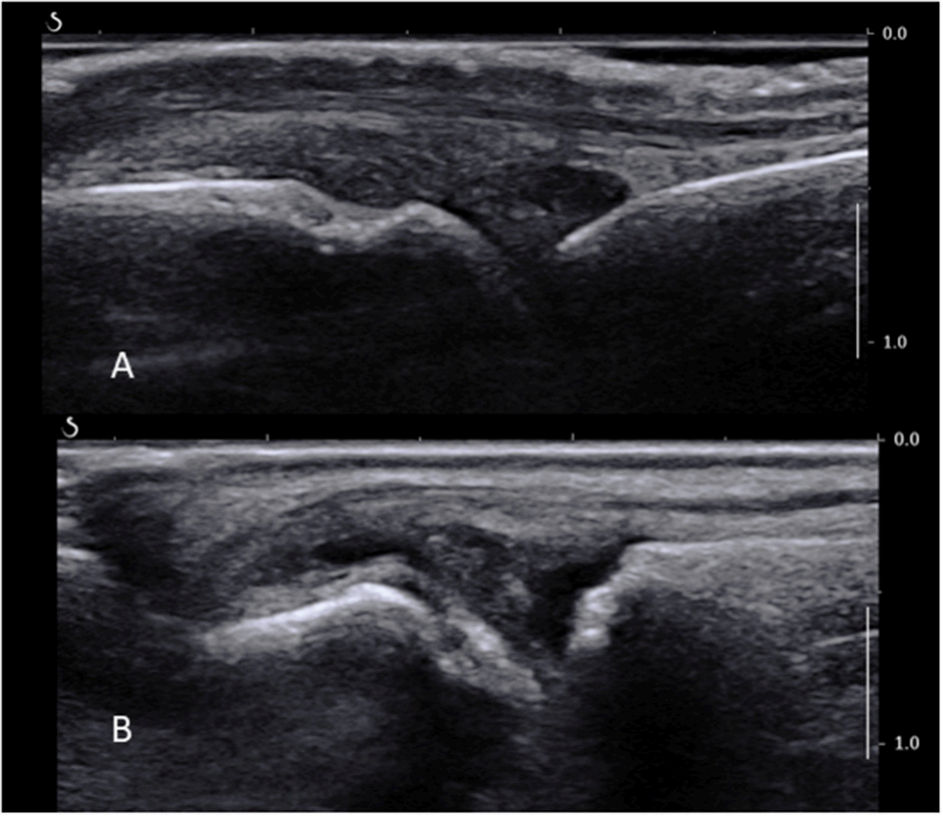
**(A)** Normal MCP joint on a longitudinal ultrasound view. Note the joint space, metacarpal head, proximal phalanx, and extensor tendon. **(B)** Long axis ultrasound scan of a fifth MTP joint showing synovial thickening proximally to the joint line overlying the metatarsal head, as well as a small amount of fluid.

Normal sonoanatomy of the joint is shown in Figure 2A. Selection of the joint to be biopsied, operator room setup and equipment, and patient positioning for the MCP joints are as per wrists. For an MTP joint biopsy, the patient is recumbent at 45° with the lower limb in flexion and the foot lying flat on the bed with special care to avoid the limb slipping during the procedure. Skin is prepped as per wrist joint with perhaps a more restricted forearm and calf area to disinfect when targeting MCP and MTP joints, respectively.

The area of interest lies proximally to the joint line and metacarpal or –tarsal head, where most of the ST is found in MCPs and MTPs (Figure 2B). A high-frequency US transducer, such as a hockey stick, should be used. The digital vessels and nerves should be identified and a path of entry to the synovium planned accordingly; this is usually dorsal to the neurovascular bundle. The MCP and MTP joints can be accessed from either their medial or lateral sides depending on the room setup and personal preference, bearing in mind that it is much more comfortable to avoid working over the patient's opposite hand or foot. The probe is placed over the area to biopsy on a short axis, and the skin and joint capsule are injected with a small volume of local anesthetic, e.g., lidocaine 1% 1 ml through a subcutaneous needle. A fine biopsy needle (16G or smaller) is used. It is inserted as the local anesthetic needle in the area of interest lying between the bone and the extensor tendon. Care should be taken not to apply too much pressure to penetrate the joint capsule, as this may lead to the needle passing through the capsule and exiting on the opposite site of the joint and finger or toe. Counter-balancing pressure by keeping a continuous contact with the skin with one or two fingers may provide more stability. Another caveat is the width of the hypertrophied synovium compared to the needle throw. This should be assessed before the procedure proceeds, as the throw (usually 10 mm) is sometimes wider than the area of ST itself, which may cause unnecessary soft tissue and/or skin damage. In this case, an alternative biopsy site must be considered. The rest of the procedure and post-procedure care follow the general rules described before.

## PIP and DIP Joints

**Figure 3:**
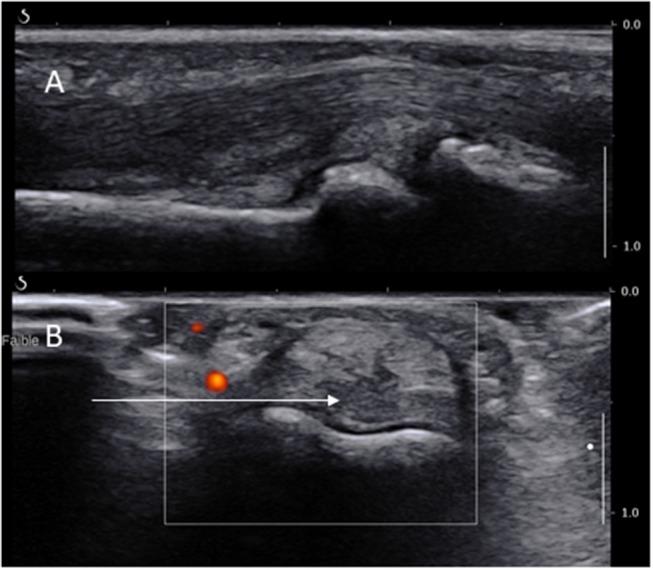
Long **(A)** and short **(B)** axis views of a PIP joint showing the long axis of the needle path (arrow) dorsally to the digital artery.

These joints are much smaller and certainly not the favored biopsy site. However, they are preferentially affected in some cases of IA and non-inflammatory conditions such as osteoarthritis. Their approach is identical to the MCP and MTP joints with two major differences with regards to pain control and entry site. The local anesthetic may be administrated through a ring block, procedure in which an anesthetic solution is injected into the base of a finger or toe thus blocking all four digital nerves (which is more time-consuming, therefore should be done after the disinfection and before the rest of the equipment is fully prepared). However, some experts report a good efficacy of a local anesthetic injection of the joint as per MCPs. The ST is more prominent on the volar side of the joint (Figure 3), just proximal to the joint line and over the distal part of the phalanx. The US probe is hence placed on the volar side of the joint and the needles are introduced medially or laterally on a short axis view deeply to the flexor tendons while avoiding neurovascular structures (see Figure 3A). It is helpful to separate the finger (or toe) from the rest by placing some sterile gauze perpendicular to its axis.

## Tendon Sheaths

Tendon sheaths can also be biopsied in the same way as any other synovial site, provided that they demonstrate at least moderate tenosynovitis and extra care is taken to avoid damaging the tendon itself. Entry site and technique (short or long axis approach) must be individualized to the specific condition and location.

## Conclusion

UGSB of the small joints is of particular interest in the setting of IA and can be safely performed with basic sonoanatomy knowledge provided that the overarching principles of tissue sampling are respected.

## Author Contributions

IL, SK, and LM contributed to the conception, drafting, and revision of the work. All authors approve its content for publication and take responsibility for its accuracy and integrity.

### Conflict of Interest Statement

The authors declare that the research was conducted in the absence of any commercial or financial relationships that could be construed as a potential conflict of interest.
